# Risk of Tobacco Smoking and Consumption of Energy Drinks on Obesity and Central Obesity Among Male University Students

**DOI:** 10.7759/cureus.21842

**Published:** 2022-02-02

**Authors:** Majdeddin Mohammed Ali, Maroun Helou, Mahdi Al-Sayed Ahmad, Rayyan Al Ali, Basma Damiri

**Affiliations:** 1 Department of Medicine, An-Najah National University, Nablus, PSE; 2 Department of Medicine and Health Sciences/Drugs and Toxicology Division, An-Najah National University, Nablus, PSE

**Keywords:** psychostimulants, chocolate, cognitive enhancers, waist circumference, central obesity, obesity, energy drinks, cigarettes, waterpipe, smoking

## Abstract

Background

Obesity is one of the leading causes of morbidity and premature death. The prevalence of obesity and being overweight in young adulthood is increasing exponentially globally, including Palestine. Consumption of energy drinks (EDs) and tobacco smoking are highly prevalent among Palestinian young adults. Although different studies have demonstrated that the use of caffeine and tobacco products is highly prevalent among Palestinians, especially university students, the adverse effects of these products on obesity have not been thoroughly investigated.

Methodology

Male students from An-Najah National University in the West Bank were recruited to fill out a self-administrated questionnaire in this cross-sectional study conducted in 2021. Obesity was measured as total adiposity by calculating body mass index (BMI) and as central obesity by measuring waist circumference. To determine the association between obesity and ED consumption and tobacco smoking, we used adjusted multiple logistic regression models. Shapiro-Wilk’s test was used to assess the normality of the data.

Results

A total of 396 students filled the questionnaire, with a response rate of 89.4%. The prevalence of obesity and central obesity was 42% and 35.75%, respectively. The prevalence of ED consumption, cigarette smoking, and waterpipe smoking was 59.6%, 39.6%, and 43.2%, respectively. ED consumers were more likely to be cigarette smokers (odds ratio (OR) = 3.827, P < 0.001), waterpipe smokers (OR = 4.578, P < 0.001), and chocolate consumers (OR = 3.524, P = 0.001). Central obesity was associated with waterpipe smoking (OR = 2.126, P = 0.044), increased age (OR = 1.367, P = 0.001), and increased BMI (OR = 1.927, P < 0.001). On the other hand, cigarette smokingincreased the risk of being underweight (OR = 6.255, P = 0.012), and ED consumption decreased the risk of being obese (OR = 0.183, P = 0.017).

Conclusions

Waterpipe smoking was a risk factor for increased central obesity, and ED consumption was associated with decreased; however, smoking was associated with the underweight BMI category. Given the undesirable health consequences of consuming the closely associated tobacco and EDs, stricter measures are needed to prevent access to these products.

## Introduction

Cardiovascular disease (CVD) is the leading cause of mortality among Palestinians [1]. Obesity and tobacco smoking (cigarettes and waterpipes) have been recognized as major risk factors in the pathogenesis of CVD, diabetes mellitus (DM), hypertension (HTN), and metabolic syndrome, which lead to an increase in all-cause mortality [2-6]. Individuals with CVD risk factors such as DM type II, HTN, and smoking are exposed to adverse health outcomes at lower levels of obesity [6]. Overweight and obese individuals exhibit chronic disease risk factors, developing diseases and consequent increase in all-cause mortality at a younger age [2].

Body mass index (BMI) is a measure of overall adiposity, while waist circumference (WC) is a measure of central adiposity [7]. Continuing to use BMI as a sole indicator of cardiometabolic risk is insufficient and should not be used solely for clinical assessment [7-9]. Recent studies have shown that central obesity can be a more informative marker than BMI [10,11]. Moreover, it is a prerequisite for metabolic syndrome diagnosis based on the available data for Palestine; therefore, European data for WC was used as recommended by the International Diabetic Federation (IDF) criterion. WC has been recognized as an independent risk factor for cardiometabolic diseases and is a better predictor of cardiovascular risk than overall obesity [8,10,11].

Obesity risk factors include, but are not limited to, poor diet and sedentary lifestyle [12,13]. A common thought among people is that cigarette and waterpipe smoking can be used for weight control [14-19]. Moreover, consuming excessive amounts of caffeine-containing fluids can serve as a weight control method by masking hunger, aiding in purging behavior, or increasing energy levels [20,21]. On the other hand, excessive sugar consumption from sugary drinks can lead to weight gain and obesity [6]. Energy drinks (EDs), beverages that contain high levels of caffeine and sugar along with other ingredients such as taurine, herbal extracts, and vitamin B, are purported to provide consumers with extra energy [22-25]. Therefore, whether ED consumption increases the risk of obesity remains uncertain [6,20,21]. Despite the uncertainty regarding their effects, ED and tobacco products have recently gained prominence in the young adult market in the West Bank. Their use tends to be higher among university students under cogitative and performative demands [26-29]. Most Palestinians start ED consumption at an early age (mean age of 15 years), noting that its consumption is associated with tobacco smoking [29].

The prevalence of being overweight and obese in young adulthood is increasing exponentially globally, including Palestine [4,30-34]. Recognizing obesity-related risk factors can help reduce the risk of obesity-related disorders. Although ED consumption and tobacco smoking are highly prevalent among Palestinians, their effect on obesity has not been investigated. This study examined the association between ED consumption and tobacco smoking and obesity among university students (18-24 years) in the West Bank, Palestine. This study is a part of ongoing studies that aim to gain a comprehensive understanding of the extent of the cognitive enhancers and psychoactive substances used among Palestinians in different target groups. Tobacco smoking, ED consumption, and psychoactive drug use are a growing problem in the West Bank. The increasing prevalence of their use and their association with negative behaviors and adverse health outcomes on Palestinians had gained considerable attention. ED consumption among male students is of particular interest. A comprehensive understanding of the risk factors associated with the increased prevalence of obesity is essential for targeted prevention strategies.

## Materials and methods

Study design, setting, sample size, and sampling technique

Tobacco smoking and ED consumption are a growing problem in the West Bank, Palestine, particularly among males. Male Palestinian students from An-Najah National University (ANNU) university were recruited to participate in a cross-sectional study conducted in 2020. The students were recruited through social media and flyers. A multistage-stratified sampling technique was used. The students were stratified according to their curricular year. Subsequently, the students were categorized according to their academic fields (50% medical and health sciences students and 50% non-medical students). The required sample size was 356 students based on a margin of error of 5% and a confidence level of 95%. Male students (N = 474) were invited to participate in the first stage of the study. Students were included if they were undergraduate students from ANNU and excluded if they had any disease or took any medication that could affect body weight. In the second stage, informed consent was obtained, anthropogenic measurements were taken, and the questionnaire was filled out.

Study tool and validity

Bodyweight was measured using a calibrated digital scale to the nearest 0.1 kg with minimal clothes and without shoes. A wall meter was used to measure standing height without shoes to the closest 0.1 cm. BMI was calculated by weight (kg)/height (m) squared. A non-elastic tape was used to assess WC to the nearest 1.0 cm. These measurements were made in compliance with the recommendations of the World Health Organization [11,33,35]. For WC values, European data were used as recommended by the IDF criterion [33]. Based on the IDF criterion, central obesity is defined as WC of ≥94 cm in men [33]. WC was measured midway between the inferior margin of the thoracic cage and the superior border of the iliac crest during minimal inspiration. The BMI (kg/m^2^) was divided into the following four categories: underweight (≥18.50), normal weight (18.5-24.99), overweight (25.0-29.99), and obese (≥30) [36]. Measurement instruments for accuracy and precision assessment have been applied and published [33,37]. A previous study verified and implemented the questionnaire to quantify tobacco smoking and ED consumption, practice, and patterns [38]. In this study, the “current user” status applied to students who had used the product at least once in the last 30 days [39]. The caffeine content of the most popular ED in the West Bank, Palestine, as labeled on the cans, ranges from 7.50 to 32.00 mg/100 mL (mean of 24.80 mg/100 mL), while the sugar content ranges from 10.00 to 11.80 g/100 mL (mean of 10.86 g/100 mL). Other caffeine sources studied in this study included black tea, coffee and its derivatives, and chocolate.

Ethical considerations

We received ethical approval from the Institutional Review Board at ANNU. All students provided a written signed informed consent before participation. The privacy and confidentiality of all participants were assured.

Statistical analysis

All analyses were performed using SPSS Statistics for Mac, version 22 (IBM Corp., Armonk, NY, USA). Shapiro-Wilk test was used to assess data normality. The means between the two groups were evaluated using the independent samples t-test. Associations between different general characteristics and outcomes were assessed using Pearson’s chi-square test and Fisher’s exact test. Adjusted multiple logistic regression analysis assessed the relative risk by generating the odds ratios (ORs) and 95% confidence intervals (CIs) for explanatory factors. All comparisons were made at a significance level (P-value) of less than 0.05.

## Results

General characteristics and anthropometric measurements of participants

The response rate was high (89.4%, n = 424). Four students were excluded, four students refused to allow the measurement of WC, and 20 partially completed questionnaires were discarded. The final number of participants was 396 students; 29.8% studied medicine, 31.3% studied health sciences, and 38.9% were in the non-health sciences programs. The prevalence of increased WC was 35.6% and obesity (BMI ≥25) was 42.2%, of which 28.8% were overweight and 13.4% were obese (Table 1).

**Table 1:** General characteristics and anthropometric measurements of participants. IQR: interquartile range

		n (%)
Locality	City	186 (47.0)
	Village	194 (49.0)
	Camp	16 (4.0)
Work status	Yes	93 (23.5)
	No	303 (76.5)
Academic specialty	Medicine	118 (29.8)
	Health sciences	124 (31.3)
	Others	154 (38.9)
Body mass index	Underweight	22 (5.6)
	Normal weight	207 (52.3)
	Overweight	114 (28.8)
	Obese	53 (13.4)
High waist circumferences	Yes	141 (35.6)
	No	255 (64.4)
Anthropometric measurements		Median ± IQR
	Weight	75.30 ± 21.37
	Height	177.00 ± 8.00
	Body mass index (kg/m^2^)	24.04 ± 6.17
	Waist circumference (cm)	84.75 ± 17.00

Prevalence and pattern of cigarette and waterpipe smoking, energy drinks, coffee, black tea, and chocolate consumption

The prevalence of ED consumption was high (59.6%), with 19.2% using it daily. The prevalence of other substances was as follows: waterpipe smoking at 43.2%, cigarette smoking at 39.6%, coffee at 85.6%, chocolate consumption at 86.1%, and black tea intake at 83.6% (Table 2).

**Table 2:** The prevalence, pattern of use, and initiation age of tobacco, energy drinks, coffee, black tea, and chocolate consumption among students.

Tobacco and caffeine products	Practice n (%)	Pattern of use n (%)					Initiation age in years, Median (Q1, Q3)
		Daily	Several times weekly	Several times monthly	Several times yearly	Ex-user	
Cigarettes	157 (39.6%)	106 (67.9)	20 (12.8)	17 (10.9)	9 (5.8)	4 (2.6)	17.00 (15.0, 18.63)
Waterpipe	171 (43.2)	53 (30.6)	29 (16.8)	53 (13.4)	28 (16.2)	10 (5.8)	17.00 (15.0, 18.00)
Energy drinks	236 (59.6)	46 (19.2)	80 (33.5)	71 (29.7)	32 (13.4)	10 (4.2)	16.00 (14.0, 18.00)
Coffee	339 (85.6)	180(56.8)	88 (27.8)	37 (11.7)	12 (3.8)	0 (0.0)	15.00 (12.0, 17.00)
Black tea	331 (83.6)	134(43.8)	105 (34.3)	50 (16.3)	15 (4.9)	2 (0.7)	10.00 (6.0, 12.00)
Chocolate	341 (86.1)	110(34.0)	140 (35.4)	62 (19.1)	7 (2.2)	5 (1.5)	6.00 (4.0, 9.25)

Multiple logistic regression models

Model 1: Risk Factors Associated With Energy Drinks Consumption

ED consumption was associated with cigarette smoking (OR = 3.827, P < 0.001), waterpipe smoking (OR = 4.578, P < 0.001), and chocolate consumption (OR = 3.524, P = 0.001) (Table 3).

**Table 3:** Adjusted multiple logistic regression for the risk factors associated with consumption of energy drinks. Note: ^a^: Reference category is No; ^b^: reference category is non-health science specialties. *Significant at P < 0.05.

Energy drinks consumption (Yes)^a^		Model 1		
	Variable	Odds ratio	95% confidence interval	P-value
Age		0.936	0.819–1.070	0.33
Cigarette smoking^a^	Yes	3.827	2.242–6.530	<0.001*
Waterpipe smoking^a^	Yes	4.578	2.777–7.546	<0.001*
Coffee consumption^a^	Yes	1.691	0.851–3.359	0.13
Chocolate consumption^a^	Yes	3.524	1.687–7.362	0.001*
Black tea consumption^a^	Yes	0.828	0.410–1.674	0.60
Work^a^	Yes	0.732	0.406–1.317	0.30
Academic specialty^b^	Medicine	0.924	0.516–1.653	0.79
	Health sciences	0.846	0.471–1.521)	0.58

Model 2: Risk Factors Associated With Central Obesity

Adjusted multiple logistic regression for the association between tobacco and ED consumption and increased central obesity (WC) revealed that the risk of increased central obesity was associated with waterpipe smoking (OR = 2.143, P = 0.042), increased age (OR = 1.366, P = 0.001), and increased BMI (OR = 1.924, P < 0.001) (Table 4).

**Table 4:** Adjusted multiple logistic regression for factors associated with increased central obesity (waist circumference). Note: ^a^: Reference category is No. *Significant at P < 0.05. WC: waist circumference

Central obesity (increased WC) (Yes)^a^		Model 2		
	Variable	Odds ratio	95% confidence interval	P-value
Energy drink consumption^a^	Yes	1.475	0.690–3.153	0.32
Cigarette smoking^a^	Yes	0.940	0.464–1.903	0.86
Waterpipe smoking^a^	Yes	2.143	1.029–4.461	0.042*
Coffee consumption^a^	Yes	0.430	0.169–1.095	0.08
Chocolate consumption^a^	Yes	0.626	0.240–1.632	0.34
Black tea consumption^a^	Yes	1.012	0.402–2.549	0.98
Age		1.366	1.145–1.629	0.001*
Body mass index		1.924	1.682–2.202	<0.001*

Model 3: Risk Factors Associated With Variable Body Mass Index Categories

Adjusted multiple logistic regression model for the association between the use of tobacco and caffeine products and BMI revealed that cigarette smoking increased the risk of being underweight (OR = 6.255, P = 0.012), and ED consumption decreased the risk of being obese (OR = 0.183, P = 0.017). In addition, increased central obesity was associated with increased BMI (P < 0.001) (Table 5).

**Table 5:** Adjusted multiple logistic regression of body mass index associated with tobacco smoking and consumption of caffeine products. Note: ^#^: Reference category is normal weight; ^a^: reference category group is No.*Significant at P < 0.05.

Body mass index category^#^		Model 3			
		Variable	Odds ratio	95% confidence interval	P-value
Underweight	Energy drinks consumption^a^	Yes	0.336	0.093–1.213	0.10
	Cigarette smoking^a^	Yes	6.255	1.498–26.125	0.012*
	Waterpipe smoking^a^	Yes	0.552	0.170–1.785	0.32
	Coffee consumption^a^	Yes	0.809	0.135–4.862	0.82
	Chocolate consumption^a^	Yes	0.148	0.202–6.520	0.88
	Black tea consumption^a^	Yes	0.929	0.199–4.326	0.93
	Age		0.710	0.505–0.998	0.049*
	Waist circumference		0.768	0.679–0.867	<0.001*
Overweight	Energy drinks consumption^#^	Yes	1.195	0.548–2.604	0.66
	Cigarette smoking^a^	Yes	0.841	0.398–1.778	0.65
	Waterpipe smoking^a^	Yes	0.527	0.243–1.139	0.10
	Coffee consumption^a^	Yes	1.374	0.542–3.483	0.50
	Chocolate consumption^a^	Yes	2.267	0.777–6.613	0.13
	Black tea consumption^a^	Yes	0.400	0.149–1.074	0.07
	Age		0.927	0.765–1.123	0.44
	Waist circumference		1.352	1.265–1. 446	<0.001*
Obese	Energy drinks consumption^a^	Yes	0.183	0.045–0.737	0.017*
	Cigarette smoking^a^	Yes	0.832	0.228–3.039	0.78
	Waterpipe smoking^a^	Yes	0.307	0.081–1.161	0.08
	Coffee consumption^a^	Yes	1.121	0.208–6.033	0.89
	Chocolate consumption^a^	Yes	2.192	0.391–12.294	0.37
	Black tea consumption^a^	Yes	0.420	0.083–2.117	0.29
	Age		0.798	0.588–1.083	0.15
	Waist circumference		1.810	1.613–2.032	<0.001*

## Discussion

Obesity is a common disease of clinical and public health importance [2,3,6]. However, studies concerning obesity and central obesity among Palestinians are scarce [40-43]. This study aimed to investigate the risk of tobacco smoking and ED consumption and obesity among Palestinian university students from ANNU. Several remarkable findings were noted in this study. First, the overall prevalence of obesity (42.2%) and central obesity (35.6%) was higher than the anticipated prevalence based on previous studies in the West Bank [33,42,44] and globally [45,46]. These results demonstrated that the prevalence of obesity has increased in recent decades, indicating that the response of society and the healthcare system to this epidemic has been insufficient. Moreover, excess body weight is the sixth most significant risk factor contributing to the global disease burden [47].

Studies on obesity and caffeine consumption have reported diverse outcomes [6,20,21]. A recently published meta-analysis concluded that coffee consumption might be correlated with lower obesity, especially among males [48]. In this study, consumption of caffeine products was highly prevalent among university students and linked to a lower risk of obesity. In a previous study, ED consumption was associated with weight loss attempts, poor body image, and unhealthy weight loss behaviors [21]. In contrast to a local study among adolescent refugees [34], ED consumption was not associated with increased central obesity. This discrepancy in results between adolescents and young adults can be related to the adolescence period or the amount of ED consumption, which was not tested in this study. Therefore, there is a need to test if the amount of ED consumption is related to the variable results of obesity and central obesity.

A previous study reported that coffee and chocolate consumption was associated with decreased risk of eating disorders only among males [34]. Obsessive chocolate eaters scored higher on drive measures for thinness, body dissatisfaction, bulimia, and binge eating disorders [49,50]. No association between chocolate consumption and obesity was observed in this study. Interestingly, ED consumption was associated with chocolate consumption, and the lower risk of obesity among ED consumers was likely due to their concurrent chocolate consumption.

In this study, most students smoked daily (67.9%). In agreement with other studies, an inverse relationship between smoking and body weight has been documented [14-19,51,52]. This study concluded that cigarette smoking is linked to being underweight. For the overweight category, decreased BMI is a risk factor for health/cardiorespiratory fitness [53]. However, the effect of smoking on body weight is not clear and can be attributed to several mechanisms, such as changes in dietary intake, physical activity, and metabolic rate [15], and is associated with the risk of metabolic syndrome [16]. Therefore, tobacco use is associated with a risk of low BMI in this population. Furthermore, in agreement with other studies, smoking was associated with ED consumption, which could worsen the problem. The higher risk of being underweight among smokers can be attributed to concurrent ED consumption [29,54-56].

In this study, waterpipe smoking was more common than cigarette smoking, with a prevalence of 43.2%. The higher rate of waterpipe smoking is a growing concern and can be attributed to the influence of urbanization on social life, social media, and the fact that waterpipe smoking is becoming more culturally and socially accepted among young adults. Waterpipes deliver 56-fold higher smoke volume compared to cigarettes, with an overlapping toxicant/chemical profile to conventional cigarettes [57]. Due to the overlapping toxicant and chemical profile to conventional cigarettes, the effects of waterpipe smoke on the cardiovascular system are thought to be comparable to those of conventional cigarettes [58]. Although an inverse relationship between cigarette smoking and overall obesity was observed, consistent with other local and global studies, waterpipe smoking was linked to a higher risk of increased central obesity in this study [34,59]. Furthermore, waterpipe smoking was linked to an increased risk of central obesity and dyslipidemia among young Palestinian adults and adolescents, as reported by a few Palestinian studies [34]. These disorders increase the risk of developing metabolic syndrome and thrombosis [11]. In addition, Palestinian schoolchildren with increased central obesity show a significant increase in the clustering of metabolic abnormalities [40]. These findings support the notion that central obesity can be more informative than BMI and a better predictor of cardiovascular risk than overall obesity [10,11]. Moreover, continuing to use BMI as a sole indicator of cardiometabolic risk is insufficient, and it should not be used solely for clinical assessment [7-9]​​​​​​​. However, further studies are needed to understand its negative health consequences and the mechanisms inducing such effects. Overall, the results of this study support the notion that waterpipe smoking is indeed detrimental to central obesity. Thus, awareness and control of waterpipe smoking should be more robust and systematic.

This study has some limitations. ED consumption and the number of cigarettes smoked per day were not tested in this study. Moreover, the lack of previous local research on obesity and central obesity associated with tobacco and caffeine product consumption was one of the most important constraints in predicting a change in obesity and central obesity in university students. More follow-up research is required. This study is the first to link ED consumption and tobacco smoking with obesity among Palestinians. This study can increase the awareness among young adults regarding the health effects of EDs on the cardiovascular system, which can lead to better health outcomes and a possible decrease in the prevalence of CVDs. Moreover, the findings of this study can influence public opinion and lead to restrictive governmental measures toward their consumption.

## Conclusions

This study provided insights into the adverse health effects of caffeine and tobacco products on obesity among university students. The findings support the notion that waterpipe smoking is indeed detrimental to central obesity. The high prevalence of ED consumption and tobacco smoking and the association between the two practices among university students is problematic. Given the health consequences of their use, policy initiatives to prevent the initiation of these products and limit access to them warrant attention. Tobacco control interventions and research are recommended along with nutrition research to improve the overall health status of the population. Incorporating EDs into drug education programs may be effective.
